# Thermochemistry of Solution, Solvation, and Hydrogen Bonding of Cyclic Amides in Proton Acceptor and Donor Solvents. Amide Cycle Size Effect

**DOI:** 10.3390/molecules26051411

**Published:** 2021-03-05

**Authors:** Ilnaz T. Rakipov, Artem A. Petrov, Aydar A. Akhmadiyarov, Artashes A. Khachatrian, Timur A. Mukhametzyanov, Boris N. Solomonov

**Affiliations:** Department of Physical Chemistry, Kazan Federal University, Kremlevskaya 18, Kazan 420008, Russia; dobriy_letchik@mail.ru (A.A.P.); aydaru@ya.ru (A.A.A.); AAHachatryan@kpfu.ru (A.A.K.); timur.mukhametzyanov@kpfu.ru (T.A.M.); boris.solomonov@kpfu.ru (B.N.S.)

**Keywords:** solution enthalpies, cyclic amides, proton acceptors, proton donors, hydrogen bonding, solution calorimetry

## Abstract

In the present work, the thermochemistry of solution, solvation, and hydrogen bonding of cyclic amides in proton acceptor (B) and proton donor (RXH) solvents were studied. The infinite dilution solution enthalpies of δ-valerolactam, *N*-methylvalerolactam, ε-caprolactam, and *N*-methylcaprolactam were measured at 298.15 K. The solvation enthalpies of cyclic amides were calculated based on the measured solution enthalpies and sublimation/vaporization enthalpies from literature. The enthalpies of hydrogen bonding between cyclic amides and proton acceptor and donor solvents were then calculated as a difference between the total solvation enthalpy and the non-specific contribution. The latter was estimated via two different approaches in proton donor and proton accepting solvents. The effect of the cycle size on the strength of hydrogen bonding of the cyclic amides in solution is discussed.

## 1. Introduction

Cyclic amides (lactams) and their derivatives demonstrate elastase inhibition [1], profound antihistaminic activity [2], and a hepatoprotective effect [3]; they are researched as components of anticancer drugs [4], electrolytes for batteries [5], and as ligands for catalytic reactions [6]. The functional properties of cyclic amides are intrinsically linked with the intermolecular interactions that they realize.

The intermolecular interactions of cyclic amides were studied by different experimental and theoretical methods [7,8,9,10,11,12,13,14]. The equality of proton acceptor ability of linear and cyclic aprotic amides was shown in work [10]. The solvation thermochemistry of protic amides in various types of solvents was studied in [11]. The primary focus of the research of these works was related to the association of amides; the interaction of amides with other organic molecules was only scarcely investigated. The relationship between the structure and thermodynamic properties of the amides was not explored.

The intermolecular interactions of linear and cyclic amides in the solutions were studied following the approach based on solvation thermochemistry [15,16,17]. The proton donor properties of linear amides are defined by the number of proton donor centers in the molecule [15]. The thermochemistry of hydrogen bonding of linear and cyclic amides with proton acceptor solvents was studied in [16]. The proton donor properties of 2-pyrrolidone were shown to be more pronounced than that of the linear amides; thus, the cyclic configuration of amide enhances its proton donor properties. The intermolecular interactions of proton acceptors in the linear and cyclic amides media were also researched [17]. The hydrogen bonding enthalpies of proton acceptors in *N*-methylformamide and 2-pyrrolidone are significantly lower than the enthalpies of hydrogen bonding of amide-base systems in 1:1 complexes. The reorganization and cooperativity effects of hydrogen bonds of linear and cyclic amides in solution were also investigated in [17].

In the present work, we have analyzed the intermolecular interactions of cyclic amides in solutions following the solution calorimetry approach. The solution enthalpies of *δ*-valerolactam, *N*-methylvalerolactam and ε-caprolactam, *N*-methylcaprolactam in proton acceptor and proton donor solvents were measured at 298.15 K. The hydrogen bonding enthalpies were evaluated based on the solution calorimetry data. To calculate hydrogen bonding enthalpy, we have used two different approaches for proton acceptor and donor solvents. The effect of the cycle size on the proton donor and acceptor properties of the cyclic amides was investigated.

## 2. Experimental Part

### 2.1. Materials

*γ*-Butyrolactam, *N*-methylbutyrolactam, *δ*-valerolactam, *N*-methylvalerolactam, ε-caprolactam, and *N*-methylcaprolactam are commercial products from Sigma-Aldrich, TCI and Acros Organics (mass fraction purity 0.97–0.99). The *N*-methylbutyrolactam purified was distilled under reduced pressure. Other cyclic amides were not purified. The cyclic amides were kept over 4 Å molecular sieves before the measurements. Proton acceptor and proton donor solvents are commercial products from Sigma-Aldrich (mass fraction purity min. 0.99). They were purified according to the methods presented by work [18]. The content of impurities was measured by chromatographic analysis on Agilent 7890 B gas chromatograph. The water content was measured using Karl-Fischer titration method. Detailed information about studied samples, their purity, and water content are presented in Supplementary Materials (Table S1).

### 2.2. Solution Calorimetry

The solution enthalpies of *N*-methylbutyrolactam, *δ*-valerolactam, *N*-methylvalerolactam, *ε*-caprolactam, and *N*-methylcaprolactam were measured at standard conditions (298.15 ± 0.01 K, 0.1 MPa) using the semi-adiabatic solution calorimeter constructed in Kazan Federal University and TAM III (TA Instruments, New Castle, PA, USA) isothermal solution calorimeter. The calorimeters were tested by the dissolution of potassium chloride and 1-propanol in water [19,20]. The calorimeter and the measurement procedure were described in works [21,22]. The enthalpies of solution of cyclic amides in solvent obtained in work are collected in Supplementary Material (Table S2 and Table S3).

## 3. Result and Discussion

### 3.1. Proton Donor Properties of Cyclic Amides

In the present work, solution enthalpies of cyclic amides in acetone, acetonitrile, ethyl acetate, tetrahydrofurane and pyridine were measured at 298.15 ± 0.01 K and 0.1 MPa. The solvent selection contains ketone, nitrile, ester, and amine for studying the proton donor properties of cyclic amides. The Proton acceptor properties of the solvents are significantly different. The solution enthalpies of cyclic amides in proton acceptors are presented in Table 1. The solution enthalpies of *γ*-butyrolactam and *N*-methylbutyrolactam in proton acceptor solvents were taken from [16].

The solution enthalpies of cyclic amides in proton acceptor solvents are notably different. The solution enthalpies of NH-cyclic amides in proton acceptor solvents are more exothermic than aprotic amides. At the same time, solution enthalpies of NH-cyclic amides depend on the cycle size. To interpret the details of the interactions between cyclic amides and aprotic solvents, the contributions to the intermolecular interactions in these systems should be separated and evaluated. It is a well-known fact that solvation enthalpy is a net quantity that includes contributions of all types of interactions between the solute and solvent molecules [23]. Therefore, to analyze the contributions of intermolecular interactions, the solvation enthalpies of amides (A) in solvent (S) were calculated in accordance with Equation (1):

(1) $$\Delta_{\mathrm{solv}}H^{A/S}\;=\;\Delta_{\mathrm{soln}}H^{A/S}\;-\;\Delta_{\mathrm{cr},l}^{g}H^{A}$$

where $\Delta_{\mathrm{soln}}H^{A/S}$—solution enthalpies of cyclic amides in aprotic solvents, $\Delta_{\mathrm{cr},l}^{g}H^{A}$—vaporization (sublimation) enthalpy of cyclic amides (A) at 298.15 K.

The solvation enthalpies of cyclic amides in proton acceptor solvents are presented in Table 2.

Vaporization and sublimation enthalpies of cyclic amides used to calculate the solvation enthalpies are presented in Table S4. The solvation enthalpies of NH-cyclic amides are found to be higher than that of aprotic amides. This difference stems from the ability of NH-cyclic amides for specific interactions with aprotic solvents. However, due to the low solubility of the cyclic amides in inert solvents, many of the standard approaches used to evaluate specific interaction contributions cannot be applied. To estimate the hydrogen bonding contribution in the interaction of cyclic amides with aprotic solvents, we have used the homomorph method, which is based on the model compound [24,25,26], following the Equation (2):

(2) $$\Delta_{\mathrm{int}(\mathrm{sp})}H^{A/S}\;=\;\Delta_{\mathrm{solv}}H^{A/S}\;-\;\Delta_{\mathrm{solv}}H^{M/S}$$

where Δ_int(sp)_*H*^A/S^ is the contribution of specific interactions (hydrogen bonding in the case of cyclic amides) to the solvation enthalpy, $\Delta_{\mathrm{solv}}H^{A/S}$ is the solvation enthalpy of cyclic amides in aprotic solvents, $\Delta_{\mathrm{solv}}H^{M/S}$ is solvation enthalpy of the model compound not capable of specific interactions (M) at 298.15 K.

This approach is free of the problems related to the need for dissolution of the studied compounds in the inert solvents. Using the data from Table 2 and Equation (2) the specific interaction enthalpies of cyclic amides in proton acceptor solvents were calculated; the results are presented in Table 3. It was found that the hydrogen bond enthalpies of cyclic amides in proton acceptor solvents, calculated using Equation (2), are comparable to the hydrogen bond enthalpies of other amides estimated earlier in [16].

The values of hydrogen bond enthalpy of cyclic amides in proton acceptor solvents are sensitive to the proton acceptor ability of the solvent (β) [27,28] irrespectively of the size of the cycle in amides, see Table 3. A comparison of these values is presented in Figure S1. On the other hand, the hydrogen bond enthalpies of *δ*-valerolactam and *ε*-caprolactam are notably lower than that of *γ*-Butyrolactam in proton acceptor solvents. This is likely due to the weakening of proton donor properties of amides with increased cycle size.

### 3.2. Proton Acceptors Properties of Cyclic Amides.

The δ-valerolactam and *ε*-caprolactam slightly soluble in proton donor solvents. For this reason, to study proton acceptor properties of the cyclic amides, the solution enthalpies of *N*-methylbutyrolactam, *N*-methylvalerolactam, *N*-methylcaprolactam in chloroform and dichloromethane were measured instead, Table 4.

The studied proton donor solvents (chloroform and dichloromethane) do not exhibit proton acceptor properties and can only form hydrogen bonds of RCH…XR type. The solution enthalpies of cyclic amides in chloroform are more exothermic than in dichloromethane.

To evaluate the contribution of the specific interaction in the case of *N*-methyl substituted amides in proton donor solvents, another approach was used.

A method for the determination of non-specific solvation enthalpy was proposed earlier [23]. This approach does not rely on the “model compounds”. Instead, non-specific solvation enthalpy can be calculated parametrically by the following Equation (3):

(3) $$\begin{array}{c} \Delta_{\mathrm{HB}}H^{A/S}\;=\;\Delta_{\mathrm{soln}}H^{A/S}\;-\;(\delta_{\mathrm{cav}}h^{S}\;-\;\delta_{\mathrm{cav}}h^{C_{6}H_{12}})\;\times \;V_{X}^{A}\;-\;\Delta_{\mathrm{soln}}H^{{A/C}_{6}H_{12}}\;-\;(a_{S_{0}}\;+\;b_{S_{0}}\sqrt{\delta h^{S}})\;\times \\ \left[(\Delta_{\mathrm{soln}}H^{A/R}\;-\;\Delta_{\mathrm{soln}}H^{{A/C}_{6}H_{12}})\;-\;(\delta_{\mathrm{cav}}h^{\mathrm{So}}\;-\;\delta_{\mathrm{cav}}h^{C_{6}H_{12}})\;\times \;V_{X}^{A}\right] \end{array}$$

where $\Delta_{so\ln}H^{A/C_{6}H_{12}}$; $\Delta_{\mathrm{soln}}H^{A/R}$ are the solution enthalpies of solute A in cyclohexane and standard solvent, $\Delta_{solv}H^{A/C_{6}H_{12}}$ is the solvation enthalpy of solute A in cyclohexane, $V_{x}^{A}$ is the McGowan characteristic volume of solute A, and $\delta_{cav}h^{S}$, $\delta_{cav}h^{R}$, $\delta_{cav}h^{C_{6}H_{12}}$ are the specific cavity formation enthalpies for each solvent.

The reference solvents should not form any specific interactions (hydrogen bond) with dissolved molecules. McGowan characteristic volume can be calculated by the additive scheme. The $\delta_{cav}^{S}$ parameter was calculated from the linear dependences of solution enthalpies of n-alkanes in solvents against the characteristic volume of n-alkanes. The detailed description of the approach and the examples of determining the contributions of non-specific intermolecular interactions of different solutes in solvents are presented in earlier works [14,15,22,29,30,31,32]. The specific relative cavity formation enthalpies of cyclohexane and benzene are 5.02 10^2^ kJ∙cm^−3^ and 1.42∙10^2^ kJ∙cm^−3^ [23], respectively.

When a non-specific interaction contribution is calculated, the solute-solvent hydrogen bond enthalpies can be calculated by subtracting the non-specific contribution from the solvation enthalpy. This approach was successfully applied to calculate solute-solvent hydrogen bond enthalpies in organic non-electrolyte solvents [33] as well in ionic liquids [34].

The hydrogen bonding enthalpies of *N*-methylbutyrolactam, *N*-methylvalerolactam, *N*-methylcaprolactam in proton donors were calculated using Equation (3). The enthalpies of solution of *N*-methylbutyrolactam, *N*-methylvalerolactam, *N*-methylcaprolactam in benzene and cyclohexane are presented in Table S4. The hydrogen bonding enthalpies (specific contribution to the solvation enthalpy) of the cyclic amides in proton donor solvents are presented in Table 5.

The proton acceptor properties of cyclic amides are not affected by the cycle size. At the same time, the enthalpy of specific interaction of *N*,*N*-dimethylformamide in chloroform is notably smaller (−13.1 kJ∙mol^−1^) [15]. Thus, cyclic amides show much greater proton acceptor properties compared to linear amides.

## 4. Conclusions

In the present work, the proton donor and proton acceptor properties were studied in the range of the linear amides. To evaluate the hydrogen bond enthalpies between the amide and the solvents, the solution enthalpies of *δ*-valerolactam, *N*-methylvalerolactam, *ε*-caprolactam, and *N*-methylcaprolactam in proton acceptor and proton donor solvents were measured at 298.15 K by solution calorimetry method. Solvation enthalpies of cyclic amides were calculated using the measured solution enthalpies and literature values of vaporization and sublimation enthalpies. The hydrogen bond enthalpies were calculated in proton acceptor and donor solvents using the applicable approach. It was found that the proton donor properties of cyclic amides decrease with the increase of the cycle sizes. On the other hand, proton acceptor properties do not depend on the cycle size.

## Figures and Tables

**Table 1:** Solution enthalpies of *γ*-butyrolactam (Bu), *N*-methylbutyrolactam (NMBu), *δ*-valerolactam, (Va), *N*-methylvalerolactam (NMVa) and *ε*-caprolactam (Ca), *N*-methylcaprolactam (NMCa) in proton acceptor solvents, (kJ·mol^−1^, *T* = 298.15 *K*, *P* = 0.1 MPa) *^a^*.

Solvent (S)	Bu	NMBu	Va	NMVa	Ca	NMCa
Acetone	3.70 ± 0.17 *^b^*	−1.19 ± 0.05 *^b^*	13.91 ± 0.13	1.5 ± 0.03	20.01 ± 0.18	1.01 ± 0.02
Acetonitrile	5.43 ± 0.01 *^b^*	−1.68 ± 0.01 *^b^*	14.04 ± 0.41	0.85 ± 0.05	20.37 ± 0.05	0.44 ± 0.02
Ethyl acetate	5.92 ± 0.07 *^b^*	−0.97 ± 0.03 *^b^*	14.81 ± 0.19	2.31 ± 0.11	20.92 ± 0.09	1.60 ± 0.02
Tetrahydrofuran	5.10 ± 0.05 *^b^*	1.58 ± 0.01 *^b^*	14.00 ± 0.09	1.58 ± 0.10	19.18 ± 0.14	1.37 ± 0.02
Pyridine	−0.29 ± 0.03 *^b^*	−2.18 ± 0.06 *^b^*	8.02 ± 0.08	−2.90 ± 0.04	13.86 ± 0.08	−2.54 ± 0.02

*^a^* Standard uncertainties u are u(T) = 0.01 K, u(p) = 10 kPa. *^b^* Enthalpies of solution taken from work [16].

**Table 2:** Solvation enthalpies of *γ*-butyrolactam (Bu), *N*-methylbutyrolactam (NMBu), *δ*-valerolactam, (Va), *N*-methylvalerolactam (NMVa) and *ε*-caprolactam (Ca), *N*-methylcaprolactam (NMCa) in proton acceptor solvents, and evaporation enthalpies of cyclic amides (kJ·mol^−1^, *T* = 298.15 *K*, *P* = 0.1 MPa) *^a^*.

Solvent (S)	Bu	NMBu	Va	NMVa	Ca	NMCa
Acetone	−70.0 ± 1.3	−57.6 ± 0.6	−64.8 ± 1.3	−58.4 ± 0.4	−67.9 ± 0.6	−62.0 ± 0.2
Acetonitrile	−68.3 ± 1.3	−58.1 ± 0.6	−64.7 ± 1.4	−59.1 ± 0.4	−67.5 ± 0.6	−62.6 ± 0.2
Ethyl acetate	−67.8 ± 1.3	−57.4 ± 0.6	−63.9 ± 1.3	−57.6 ± 0.4	−67.0 ± 0.6	−61.4 ± 0.2
Tetrahydrofurane	−68.6 ± 1.3	−54.8 ± 0.6	−64.7 ± 1.3	−58.3 ± 0.4	−68.7 ± 0.6	−61.6 ± 0.2
Pyridine	−74.0 ± 1.3	−58.6 ± 0.6	−70.7 ± 1.3	−62.8 ± 0.4	−74.0 ± 0.6	−65.5 ± 0.2
$\Delta_{\mathrm{cr},l}^{g}H^{A}$	73.7 ± 1.3 *^b^*	56.4 ± 0.6 *^b^*	78.7 ± 1.3 *^b^*	59.9 ± 0.4 *^b^*	87.9 ± 0.6 *^b^*	63.0 ± 0.2 *^b^*

*^a^* Standard uncertainties u are *u*(T) = 0.01 K, *u*(p) = 10 kPa. *^b^* Enthalpies of vaporization and sublimation ($\Delta_{\mathrm{cr},l}^{g}H^{A}$) taken from Table S4.

**Table 3:** Hydrogen bonding enthalpies of *γ*-butyrolactam, *δ*-valerolactam and *ε*-caprolactam in proton acceptor solvents (S), (kJ·mol^−1^, *T* = 298.15 *K*, *P* = 0.1 MPa) *^a^*.

Solvent (S)	*γ*-Butyrolactam	*δ*-Valerolactam	*ε*-Caprolactam	β
Acetone	−12.4 (−11.5) ^b^	−6.4	−5.9	0.48
Acetonitrile	−10.2 (−8.9) ^b^	−5.6	−5.0	0.31
Ethyl acetate	−10.4 (−10.4) ^b^	−6.3	−5.6	0.45
Tetrahydrofurane	−13.8 (−13.2) ^b^	−6.4	−7.1	0.55
Pyridine	−15.4 (−6.1) ^b^	−7.9	−8.5	0.64

*^a^* Standard uncertainties u are *u*(T) = 0.01 K, *u*(p) = 10 kPa. *^b^* Enthalpies of hydrogen bonding of *γ*-butyrolactam in solvent taken from work [16].

**Table 4:** Solution enthalpies of *N*-methylbutyrolactam, *N*-methylvalerolactam, *N*-methylcaprolactam in proton donor solvents (S), (kJ·mol^−1^, *T* = 298.15 *K*, *P* = 0.1 MPa) *^a^*.

Solvent (S)	*N*-methylbutyrolactam	*N*-methylvalerolactam	*N*-methylcaprolactam
Dichloromethane	−8.56 ± 0.05	−8.33 ± 0.05	−7.28 ± 0.04
Chloroform	−19.06 ± 0.09	−19.29 ± 0.07	−17.97 ± 0.05

*^a^* Standard uncertainties u are *u*(T) = 0.01 K, *u*(p) = 10 kPa. Uncertainties of solution enthalpies are calculated as a standard deviation of the mean.

**Table 5:** Hydrogen bonding enthalpies of *N*-methylbutyrolactam, *N*-methylvalerolactam, *N*-methylcaprolactam in proton donor solvents (S), (kJ·mol^−1^, *T*= 298.15 *K*, *P* = 0.1 MPa) *^a^*.

Solvent (S)	*N*-methylbutyrolactam	*N*-methylvalerolactam	*N*-methylcaprolactam
Dichloromethane	−4.9	−4.5	−3.9
Chloroform	−17.7	−17.8	−16.3

*^a^* Standard uncertainties u are *u*(T) = 0.01 K, *u*(p) = 10 kPa.

## Data Availability

Data are contained within the article and Supplementary Materials.

## Supplementary Materials

The following are available online. Table S1: The chemicals used in this study ^a^, Table S2: Solution enthalpies of δ-Valerolactam, N-Methylvalerolactam and ε-caprolactam, N-methyl-ε-caprolactam in proton acceptors solvents measured in this work at 298.15 K and 0.1 MPa ^a^, Table S3: Solution enthalpies of N-methylbutyrolactam, N-methylvalerolactam, N–Methyl-ε-caprolactam in proton acceptors solvents measured in this work at 298.15 K and 0.1 MPa ^a^, Table S4: Characteristic volumes of amides ($V_{X}^{A}\;\times \;10^{-2}$/cm^3^∙mol^−1^), solution enthalpies of cyclic amides in benzene ($\Delta_{\mathrm{soln}}H^{A/R}$/kJ∙mol^−1^), cyclohexane ($\Delta_{\mathrm{soln}}H^{{A/C}_{6}H_{12}}$/kJ∙mol^−1^) measured in this work at 298.15 K and 0.1 MPa ^a^, Table S5: Enthalpies of vaporization and sublimation of cyclic amides taken from literature at 298.15 K and 0.1 MPa, kJ mol−1, Figure S1: Comparison of parameter β and enthalpies of hydrogen bond of cyclic amides in proton acceptor solvents: ○-γ-butyrolactam, □-δ-valerolactam, Δ-ε-caprolactam: (1-acetonitrile, 2-ethyl acetate, 3-acetone, 4-tetrahydrofurane, 5-pyridine).

## Sample Availability

Samples of the compounds are available from the authors.
