# Supplementary material for: Thermochemistry of Solution, Solvation, and Hydrogen Bonding of Cyclic Amides in Proton Acceptor and Donor Solvents. Amide Cycle Size Effect
Source: Molecules. 2021 Mar 5;26(5):1411. doi: 10.3390/molecules26051411 (PMC7961564; doi:10.3390/molecules26051411)
Supplement: Supplementary file 1 [file molecules-26-01411-s001.pdf]

SUPPLEMENTARY MATERIAL

Thermochemistry of solution, solvation, and hydrogen bonding of cyclic amides in proton acceptor and donor solvents. Amide cycle size effect

Ilnaz T. Rakipov*, Artem A. Petrov, Aydar A. Akhmadiyarov, Artashes A. Khachatrian, Timur A. Mukhametzynov, Boris N. Solomonov.

Department of Physical Chemistry, Kazan Federal University, Kremlevskaya 18, Kazan, 420008, Russia

[1]To whom correspondence concerning the solution calorimetry experiments and hydrogen bond analysis should be addressed. E-mail: ilnaz0805@gmail.com

Table 1. The chemicals used in this study[a].

Compound	CAS number	Source	Initial mass fraction purity	Purification method	Final mass fraction purity	Mass fraction of water[b]
Acetone (l)	179124	Sigma-Aldrich	0.995	Distillation	0.997	0.0003
Acetonitrile (l)	75-05-8	Sigma-Aldrich	0.999	Distillation	0.998	0.0002
Ethyl acetate (l)	141-78-6	Sigma-Aldrich	0.995	Distillation	0.996	0.0004
Tetrahydrofuran (l)	109-99-9	Sigma-Aldrich	0.99	Distillation	0.997	0.0004
Pyridine (l)	110-86-1	Sigma-Aldrich	0.99	Distillation	0.997	0.0003
2 γ-Butyrolactam (l)	616-45-5	Sigma-Aldrich	0.99	Distillation under reduce pressure	0.998	0.0003
N-Methylbutyrolactam (l)	872-50-4	Sigma-Aldrich	0.99	Distillation under reduce pressure	0.996	0.0004
δ-Valerolactam (cr.)	675-20-7	Acros Organics	0.99	None	-	0.0008
N-Methylvalerolactam (l)	931-20-4	Sigma-Aldrich	0.99	None	-	0.0006
ε-Caprolactam (cr)	105-60-2	Acros Organics	0.99	None	-	0.0011
N–Methylcaprolactam (l)	2556-73-2	TCI	>0.97	None	-	0.0008

[a] cr. – substances under standard conditions in the solid state, (l) – substances under standard conditions in the liquid state

[b] Determined by Karl Fischer titration.

They were purified according to the methods presented by work D.D. Perrin, W.L.F. Armarego, D.R. Perrin, Purification of Laboratory Chemicals, 1980.

Table S2. Solution enthalpies of δ-Valerolactam, *N*-Methylvalerolactam and ε-caprolactam, N-methyl-ε-caprolactam in proton acceptors solvents measured in this work at 298.15K and 0.1MPa[a].

Solute (A)	Solvent (S)	m/mg [b]	b/mol·kg^{-1} × 10^{3} [c]	$\Delta_{soln}H^{A/S}$ / kJ·mol^{-1} [d]
δ-Valerolactam (cr)	Acetone (l)	37.3	2.40	13.86
δ-Valerolactam (cr)	Acetone (l)	38.3	4.86	14.03
δ-Valerolactam (cr)	Acetone (l)	37.7	7.29	14.16
δ-Valerolactam (cr)	Acetone (l)	37.2	9.68	13.57
			$\Delta_{soln}H^{A/S} = 13.91 \pm 0.13$ kJ·mol^{-1} [e]	
δ-Valerolactam (cr)	Acetonitrile (l)	37.7	2.42	13.20
δ-Valerolactam (cr)	Acetonitrile (l)	37.8	4.84	14.65
δ-Valerolactam (cr)	Acetonitrile (l)	37.1	7.23	14.82
δ-Valerolactam (cr)	Acetonitrile (l)	37.4	9.63	13.50
			$\Delta_{soln}H^{A/S} = 14.04 \pm 0.41$ kJ·mol^{-1} [e]	
δ-Valerolactam (cr)	Ethyl acetate (l)	38.0	2.12	15.05
δ-Valerolactam (cr)	Ethyl acetate (l)	40.6	4.40	14.44
δ-Valerolactam (cr)	Ethyl acetate (l)	40.1	6.64	14.55
δ-Valerolactam (cr)	Ethyl acetate (l)	40.7	8.91	15.20
			$\Delta_{soln}H^{A/S} = 14.81 \pm 0.19$ kJ·mol^{-1} [e]	
δ-Valerolactam (cr)	Tetrahydrofurane (l)	38.6	2.19	14.17
δ-Valerolactam (cr)	Tetrahydrofurane (l)	39.9	4.45	13.99
δ-Valerolactam (cr)	Tetrahydrofurane (l)	37.0	6.55	13.75
δ-Valerolactam (cr)	Tetrahydrofurane (l)	36.4	8.62	14.10
			$\Delta_{soln}H^{A/S} = 14.00 \pm 0.09$ kJ·mol^{-1} [e]	
δ-Valerolactam (cr)	Pyridine (l)	39.4	2.02	8.11
δ-Valerolactam (cr)	Pyridine (l)	37.0	3.92	7.89
δ-Valerolactam (cr)	Pyridine (l)	41.0	6.03	8.18
δ-Valerolactam (cr)	Pyridine (l)	37.3	7.95	7.88
			$\Delta_{soln}H^{A/S} = 8.02 \pm 0.08$ kJ·mol^{-1} [e]	
N-Methylvalerolactam (l)	Acetone (l)	36.4	2.05	1.46
N-Methylvalerolactam (l)	Acetone (l)	34.3	3.98	1.55
N-Methylvalerolactam (l)	Acetone (l)	33.3	5.86	1.54
N-Methylvalerolactam (l)	Acetone (l)	32.7	7.70	1.43
			$\Delta_{soln}H^{A/S} = 1.50 \pm 0.03$ kJ·mol^{-1} [e]	
N-Methylvalerolactam (l)	Acetonitrile (l)	33.6	1.89	0.74
N-Methylvalerolactam (l)	Acetonitrile (l)	33.0	3.74	0.91
N-Methylvalerolactam (l)	Acetonitrile (l)	32.8	5.59	0.95
N-Methylvalerolactam (l)	Acetonitrile (l)	36.6	7.65	0.78
			$\Delta_{soln}H^{A/S} = 0.85 \pm 0.05$ kJ·mol^{-1} [e]	
N-Methylvalerolactam (l)	Ethyl acetate (l)	34.8	1.70	2.01

N-Methylvalerolactam (l)	Ethyl acetate (l)	34.6	3.40	2.52
N-Methylvalerolactam (l)	Ethyl acetate (l)	33.7	5.05	2.44
N-Methylvalerolactam (l)	Ethyl acetate (l)	35.2	6.77	2.25
			$\Delta_{soln}H^{A/S} = 2.31 \pm 0.11$	$kJ \cdot mol^{-1}$ [e]
N-Methylvalerolactam (l)	Tetrahydrofurane (l)	34.1	1.69	1.42
N-Methylvalerolactam (l)	Tetrahydrofurane (l)	34.2	3.39	1.65
N-Methylvalerolactam (l)	Tetrahydrofurane (l)	34.1	5.09	1.83
N-Methylvalerolactam (l)	Tetrahydrofurane (l)	34.6	6.81	1.40
			$\Delta_{soln}H^{A/S} = 1.58 \pm 0.10$	$kJ \cdot mol^{-1}$ [e]
N-Methylvalerolactam (l)	Pyridine (l)	33.3	1.50	-3.01
N-Methylvalerolactam (l)	Pyridine (l)	35.7	3.10	-2.88
N-Methylvalerolactam (l)	Pyridine (l)	35.0	4.68	-2.84
N-Methylvalerolactam (l)	Pyridine (l)	36.2	6.31	-2.88
			$\Delta_{soln}H^{A/S} = -2.90 \pm 0.04$	$kJ \cdot mol^{-1}$ [e]
ε-Caprolactam (cr)	Acetone (l)	33.4	1.88	19.88
ε-Caprolactam (cr)	Acetone (l)	37.3	3.98	20.50
ε-Caprolactam (cr)	Acetone (l)	35.2	5.97	19.99
ε-Caprolactam (cr)	Acetone (l)	33.2	7.84	19.65
			$\Delta_{soln}H^{A/S} = 20.01 \pm 0.18$	$kJ \cdot mol^{-1}$ [e]
ε-Caprolactam (cr)	Acetonitrile (l)	37.5	2.11	20.29
ε-Caprolactam (cr)	Acetonitrile (l)	34.9	4.07	20.28
ε-Caprolactam (cr)	Acetonitrile (l)	36.0	6.09	20.44
ε-Caprolactam (cr)	Acetonitrile (l)	36.9	8.17	20.45
			$\Delta_{soln}H^{A/S} = 20.37 \pm 0.05$	$kJ \cdot mol^{-1}$ [e]
ε-Caprolactam (cr)	Ethyl acetate (l)	37.7	1.85	21.07
ε-Caprolactam (cr)	Ethyl acetate (l)	36.6	3.64	20.85
ε-Caprolactam (cr)	Ethyl acetate (l)	35.6	5.38	20.70
ε-Caprolactam (cr)	Ethyl acetate (l)	37.1	7.20	21.04
			$\Delta_{soln}H^{A/S} = 20.92 \pm 0.09$	$kJ \cdot mol^{-1}$ [e]
ε-Caprolactam (cr)	Tetrahydrofurane (l)	34.0	1.69	18.94
ε-Caprolactam (cr)	Tetrahydrofurane (l)	36.4	3.50	19.44
ε-Caprolactam (cr)	Tetrahydrofurane (l)	35.7	5.27	19.42
ε-Caprolactam (cr)	Tetrahydrofurane (l)	36.1	7.07	18.92
			$\Delta_{soln}H^{A/S} = 19.18 \pm 0.14$	$kJ \cdot mol^{-1}$ [e]
ε-Caprolactam (cr)	Pyridine (l)	36.8	1.66	13.68
ε-Caprolactam (cr)	Pyridine (l)	36.3	3.29	14.01
ε-Caprolactam (cr)	Pyridine (l)	33.3	4.79	13.77
ε-Caprolactam (cr)	Pyridine (l)	33.9	6.31	13.99
			$\Delta_{soln}H^{A/S} = 13.86 \pm 0.08$	$kJ \cdot mol^{-1}$ [e]
N–Methylcaprolactam (l)	Acetone (l)	36.4	1.83	0.99
N–Methylcaprolactam (l)	Acetone (l)	32.7	3.47	1.07
N–Methylcaprolactam (l)	Acetone (l)	35.7	5.26	1.00

N–Methylcaprolactam (l)	Acetone (l)	35.9	7.06	0.99
				$\Delta_{soln}H^{A/S} = 1.01 \pm 0.02$ kJ·mol^{-1} [e]
N–Methylcaprolactam (l)	Acetonitrile (l)	33.5	1.68	0.39
N–Methylcaprolactam (l)	Acetonitrile (l)	35.6	3.46	0.50
N–Methylcaprolactam (l)	Acetonitrile (l)	33.2	5.12	0.42
N–Methylcaprolactam (l)	Acetonitrile (l)	35.3	6.88	0.45
				$\Delta_{soln}H^{A/S} = 0.44 \pm 0.02$ kJ·mol^{-1} [e]
N–Methylcaprolactam (l)	Ethyl acetate (l)	35.5	1.55	1.65
N–Methylcaprolactam (l)	Ethyl acetate (l)	33.9	3.02	1.55
N–Methylcaprolactam (l)	Ethyl acetate (l)	32.8	4.45	1.56
N–Methylcaprolactam (l)	Ethyl acetate (l)	34.3	5.95	1.63
				$\Delta_{soln}H^{A/S} = 1.60 \pm 0.02$ kJ·mol^{-1} [e]
N–Methylcaprolactam (l)	Tetrahydrofurane (l)	35.7	1.58	1.33
N–Methylcaprolactam (l)	Tetrahydrofurane (l)	34.7	3.11	1.41
N–Methylcaprolactam (l)	Tetrahydrofurane (l)	32.9	4.57	1.34
N–Methylcaprolactam (l)	Tetrahydrofurane (l)	35.8	6.15	1.41
				$\Delta_{soln}H^{A/S} = 1.37 \pm 0.02$ kJ·mol^{-1} [e]
N–Methylcaprolactam (l)	Pyridine (l)	36.3	1.45	-2.50
N–Methylcaprolactam (l)	Pyridine (l)	34.0	2.81	-2.50
N–Methylcaprolactam (l)	Pyridine (l)	33.5	4.16	-2.54
N–Methylcaprolactam (l)	Pyridine (l)	33.3	5.49	-2.60
				$\Delta_{soln}H^{A/S} = -2.54 \pm 0.02$ kJ·mol^{-1} [e]

[a] Standard uncertainties *u* are *u*(T) = 0.01 K. *u*(p) = 10 kPa. Cr. – substances under standard conditions in the solid state, (l) – substances under standard conditions in the liquid state

[b] Mass of solute sample which was added in each dissolution experiment (*u(m)* = 0.1 mg)

[c] Molality of solute in solution after experiments (*u(b)* = 0.00005)

[d] Enthalpy of solution of each experiment

[e] Average enthalpy of solution. Uncertainty is calculated as a standard deviation of the mean

Table S3. Solution enthalpies of N-methylbutyrolactam, N-methylvalerolactam, N–Methyl-ε-caprolactam in proton acceptors solvents measured in this work at 298.15K and 0.1MPa[a]

Solute (A)	Solvent (S)	m/mg [b]	b/mol·kg^{-1} × 10^{3} [c]	$\Delta_{soln}H^{A/S}$ / kJ·mol^{-1} [d]
N-Methylbutyrolactam (l)	Dichloromethane (l)	36.3	1.38	-8.49
N-Methylbutyrolactam (l)	Dichloromethane (l)	34.4	2.68	-8.64
N-Methylbutyrolactam (l)	Dichloromethane (l)	35.3	4.02	-8.67
N-Methylbutyrolactam (l)	Dichloromethane (l)	34.2	5.31	-8.45
				$\Delta_{soln}H^{A/S} = -8.56 \pm 0.05$ kJ·mol^{-1} [e]
N-Methylbutyrolactam (l)	Chloroform (l)	34.1	1.16	-19.22
N-Methylbutyrolactam (l)	Chloroform (l)	37.4	2.42	-18.94
N-Methylbutyrolactam (l)	Chloroform (l)	37.6	3.69	-19.22
N-Methylbutyrolactam (l)	Chloroform (l)	36.3	4.92	-18.86
				$\Delta_{soln}H^{A/S} = -19.06 \pm 0.09$ kJ·mol^{-1} [e]

N-Methylvalerolactam (l)	Dichloromethane (l)	35.1	1.17	-8.45
N-Methylvalerolactam (l)	Dichloromethane (l)	35.6	2.35	-8.34
N-Methylvalerolactam (l)	Dichloromethane (l)	35.8	3.54	-8.27
N-Methylvalerolactam (l)	Dichloromethane (l)	35.5	4.72	-8.24
				$\Delta_{\text{soln}}H^{A/S} = -8.33 \pm 0.05\ \text{kJ}\cdot\text{mol}^{-1}$ [e]
N-Methylvalerolactam (l)	Chloroform (l)	36.4	1.08	-19.18
N-Methylvalerolactam (l)	Chloroform (l)	36.1	2.15	-19.39
N-Methylvalerolactam (l)	Chloroform (l)	36.3	3.23	-19.16
N-Methylvalerolactam (l)	Chloroform (l)	35.6	4.28	-19.41
				$\Delta_{\text{soln}}H^{A/S} = -19.29 \pm 0.07\ \text{kJ}\cdot\text{mol}^{-1}$ [e]
N–Methylcaprolactam (l)	Dichloromethane (l)	35.6	1.05	-7.21
N–Methylcaprolactam (l)	Dichloromethane (l)	33.8	2.05	-7.36
N–Methylcaprolactam (l)	Dichloromethane (l)	36.2	3.12	-7.22
N–Methylcaprolactam (l)	Dichloromethane (l)	35.9	4.18	-7.33
				$\Delta_{\text{soln}}H^{A/S} = -7.28 \pm 0.04\ \text{kJ}\cdot\text{mol}^{-1}$ [e]
N–Methylcaprolactam (l)	Chloroform (l)	35.4	0.93	18.07
N–Methylcaprolactam (l)	Chloroform (l)	34.0	1.83	17.86
N–Methylcaprolactam (l)	Chloroform (l)	32.8	2.70	18.06
N–Methylcaprolactam (l)	Chloroform (l)	35.9	3.64	17.90
				$\Delta_{\text{soln}}H^{A/S} = -17.97 \pm 0.05\ \text{kJ}\cdot\text{mol}^{-1}$ [e]

[a] Standard uncertainties *u* are *u*(T) = 0.01 K. *u*(p) = 10 kPa, (l) – substances under standard conditions in the liquid state

[b] Mass of solute sample which was added in each dissolution experiment (*u(m)* = 0.1 mg)

[c] Molality of solute in solution after experiments (*u*(*b*) = 0.00005)

[d] Enthalpy of solution of each experiment

[e] Average enthalpy of solution. Uncertainty is calculated as a standard deviation of the mean

Table S 4 Characteristic volumes of amides ($V_X^A \times 10^{-2}$ /cm³·mol⁻¹), solution enthalpies of cyclic amides in benzene ($\Delta_{\text{soln}}H^{\text{A/R}}$ / kJ·mol⁻¹), cyclohexane ($\Delta_{\text{soln}}H^{\text{A/C}_6\text{H}_{12}}$ / kJ·mol⁻¹) measured in this work at 298.15 K and 0.1 MPa[a].

Cyclic amides (A)	$V_X^A \times 10^{-2}$	$\Delta_{\text{soln}}H^{\text{A/R}}$	$\Delta_{\text{soln}}H^{\text{A/C}_6\text{H}_{12}}$
N-Methylbutyrolactam	0.8200	-1.69±0.02[b]	12.30±0.10[b]
N-Methylvalerolactam	0.9609	-1.68±0.03[c]	12.12±0.05[c]
N-Methylcaprolactam	1.1018	-1.09±0.04[c]	11.02±0.03[c]

[a] Standard uncertainties *u* are *u*(T) = 0.01 K, *u*(p) = 10 kPa. [b]

[b] taken from I.T. Rakipov, A.N. Sabirzyanov, A.A. Petrov, A.A. Akhmadiayrov, M.A. Varfolomeev, B.N. Solomonov, Thermochemistry of hydrogen bonding of linear and cyclic amides in proton acceptors media, Thermochim. Acta, 652 (2017) 34-38.

[c] Enthalpies of solution measured in this work

Table S5. Enthalpies of vaporization and sublimation of cyclic amides taken from literature at 298.15 K and 0.1 MPa, kJ·mol^{-1}.

Solute (A)	$\Delta_{ev}H^{A_i/S}$	Ref.
γ-Butyrolactam	**73.7±1.3**	[1]
N-Methylbutyrolactam	56.9	[2]
	55.8	[3]
	61.9	[4]
	57.7	[5]
	56.1	[6]
	56.8	[7]
	55.9	[7]
	55.1	[8]
	56.1	[9]
	54.3	[8]
	55.2	[10]
	54.9	[11]
	**56.4±0.6**[a]	**average**
δ-Valerolactam	**78.7±1.3**	**[12]**
N-Methylvalerolactam	60.3	[12]
	59.5	[8]
	**59.9±0.4**[a]	**average**
ε-Caprolactam	86.8	[13]
	87.5	[13]
	90.3	[14]
	87.3	[14]
	87.3	[14]
	88.3	[15]
	**87.9±0.6**[a]	**average**
N-Methylcaprolactam	**63.0±0.2**	[3]

[a]Average enthalpy of solution. Uncertainty is calculated as a standard deviation of the mean

References

[1] K.M. Morgan, D.A. Kopp, Solvent effects on the stability of simple secondary amides, J. Chem. Soc., Perkin Trans. 2, (1998) 2759-2763.

[2] K.R. Reddy, D.B.K. Kumar, G.S. Rao, P.B.S. Sri, C. Rambabu, Vapor-liquid equilibria and excess molar volumes of N-methyl-2-pyrrolidone with 2-alkoxyethanols, Fluid Phase Equilib., 336 (2012) 52-58.

[3] M. Palczewska-Tulińska, P. Oracz, Vapor pressures of 1-methyl-2-pyrrolidone, 1-methyl-azepan-2-one, and 1,2-epoxy-3-chloropropane, J. Chem. Eng. Data, 52 (2007) 2468-2471.

[4] K. Chyliński, Z. Fraś, S.K. Malanowski, Vapor-liquid equilibrium for propylene glycol + 2-(2-hexyloxyethoxy)ethanol and 1-methyl-2-pyrrolidone + 1-methoxypropan-2-ol, J. Chem. Eng. Data, 49 (2004) 18-23.

[5] B. Blanco, S. Beltrán, J.L. Cabezas, J. Coca, Phase equilibria of binary systems formed by hydrocarbons from petroleum fractions and the solvents N-methylpyrrolidone and N,N-dimethylformamide. 1. Isobaric vapor-liquid equilibria, J. Chem. Eng. Data, 42 (1997) 938-942.

[6] J. Linek, I. Wichterle, K.N. Marsh, Vapor-liquid equilibria for N-methyl-2-pyrrolidone + benzene, +toluene, +heptane, and +methylcyclohexane, J. Chem. Eng. Data, 41 (1996) 1212-1218.
[7] P. Kneisl, J.W. Zondlo, Vapor Pressure, Liquid Density, and the Latent Heat of Vaporization as Functions of Temperature for Four Dipolar Aprotic Solvents, J. Chem. Eng. Data, 32 (1987) 11-13.
[8] R.M. Stephenson, S. Malanowski, Handbook of the Thermodynamics of Organic Compounds, Elsevier, New
York,, 1987.
[9] V.I. Bludilina, A.K. Baev, V.K. Matveev, I.L. Gaidym, E.I. Shcherbina, Zh. Fiz. Khim, 53 (1979) 1052-1053.
[10] T.E. Daubert, D.R. P., Physical and Thermodynamic Properties of Pure Chemicals,, Taylor & Francis, , London, 1989.
[11] W.V. Steele, R.D. Chirico, A. Nguyen, I.A. Hossenlopp, N.K. Smith, Determination of ideal-gas enthalpies of formation for key compounds, in: AIChE Symp. Ser., 1990, pp. 138-154.
[12] M.A.V. Ribeiro Da Silva, J.I.T.A. Cabral, Thermochemistry of some derivatives of 2- and 4-piperidone, J. Chem. Eng. Data, 51 (2006) 1556-1561.
[13] D.H. Zaitsau, Y.U. Paulechka, G.J. Kabo, A.N. Kolpikau, V.N. Emel'yanenko, A. Heintz, S.P. Verevkin, Thermodynamics of the sublimation and of the vaporization of ε-caprolactam, J. Chem. Eng. Data, 51 (2006) 130-135.
[14] G.J. Kabo, A.A. Kozyro, V.S. Krouk, V.M. Sevruk, I.A. Yursha, V.V. Simirsky, V.I. Gogolinsky, Thermodynamic properties of 6-aminohexanoic lactam (ε-caprolactam), J. Chem. Thermodyn., 24 (1992) 1-13.
[15] V.L.S. Freitas, M.D.M.C. Ribeiro da Silva, Thermodynamic properties of ε-caprolactam and ε-caprothiolactam, J. Chem. Thermodyn., 132 (2019) 451-460.

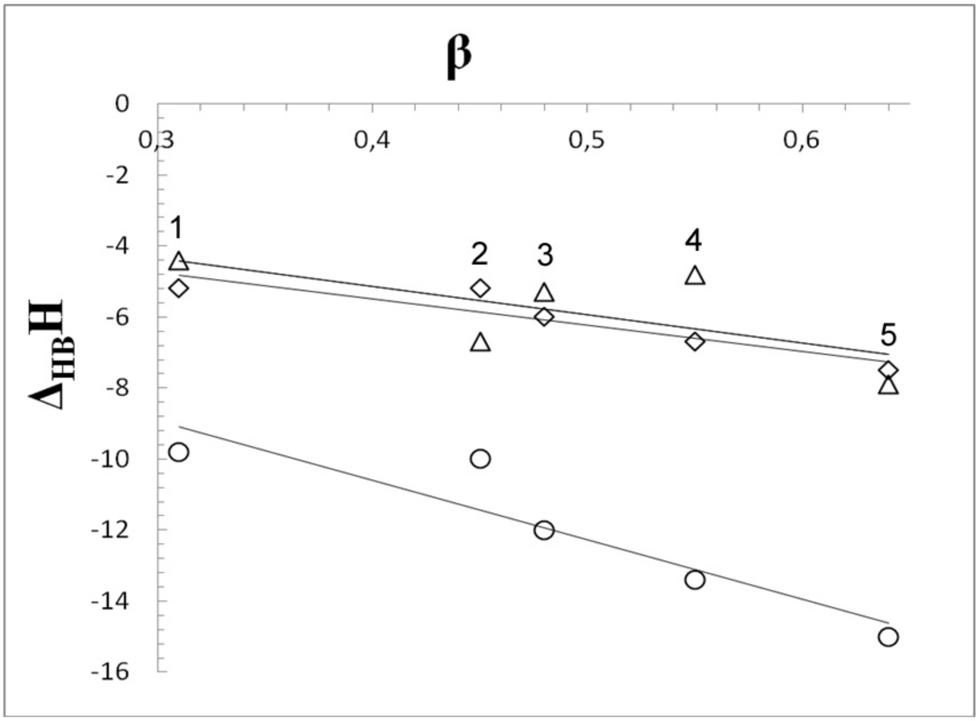

Fig .S1. Comparison of parameter β and enthalpies of hydrogen bond of cyclic amides in proton acceptor solvents: ○ - γ-butyrolactam, □ - δ-valerolactam, Δ - ε-caprolactam: (1- acetonitrile, 2-ethyl acetate, 3 - acetone, 4- tetrahydrofurane, 5- pyridine)
